# Investigations on the Performance of Shotcrete Using Artificial Lightweight Shale Ceramsite as Coarse Aggregate

**DOI:** 10.3390/ma15103528

**Published:** 2022-05-13

**Authors:** Shengjun Hou, Fuhai Li, Huiqi Tang, Tao Wen, Zhao Chen, Hao Gao

**Affiliations:** 1 Power China Roadbridge Group Co., Ltd., Beijing 100160, China; lqhousj@powerchina.cn; 2 Institute of Civil Engineering Materials, School of Civil Engineering, Southwest Jiaotong University, Chengdu 610031, China; lifuhai2007@home.swjtu.edu.cn (F.L.); huiqitang@my.swjtu.edu.c (H.T.); wentao1309@my.swjtu.edu.cn (T.W.); gaohao@my.swjtu.edu.cn (H.G.); 3 Key Laboratory of Seismic Engineering Technology of Sichuan Province, Southwest Jiaotong University, Chengdu 610031, China

**Keywords:** lightweight aggregate shotcrete, artificial lightweight shale ceramsite, chloride ion penetration resistance, contact stress, rebound model

## Abstract

In this study, a type of artificial lightweight shale ceramsite (ALSC) was used as the coarse lightweight aggregate for shotcrete (LAS), of which the mechanical properties, chloride penetration ion resistance, and rebound behavior were investigated. Based on the experimental results on compressive, tensile, and bond strength, LAS meet the strength requirements, and the replacement rate of fly ash (FA) and silica fume (SF) are suggested to be kept at ~15% and 10%, respectively, to result in the best mechanical properties of LAS. Adding FA and SF to the mixture significantly improved the chloride ion penetration resistance (CPR) of LAS because of morphology effects and secondary hydration of FA and SF that lead to a denser microstructure of the mixture. The electric flux and chloride ion migration coefficient (*D_RCM_*) of LAS decreased by 56% and 67%, respectively, with FA increasing from 0 to 10%. The electric flux and *D_RCM_* further decreased by 71% (153C) and 66% (3.24 m^2^/s), respectively, with FA increasing from 10 to 20%. As 5–10% SF was further added, the electric flux and *D_RCM_* of LAS decreased to extremely low levels; for instance, with FA = 10% and SF = 10%, *D_RCM_* = 1.61 m^2^/s, and the electric flux was too small and could be ignored. The contact stresses between aggregate and shotcrete mixtures were measured to investigate the rebound trend of ALSC in shotcrete. According to the analyses of the theoretical model of the rebound behavior of aggregate in shotcrete proposed by Armelin and Banthia, because of the reduced contact stresses between ALSC and mortar and the smaller density of LAS compared with normal-weight aggregate, the rebound rate of ALSC was about half of that of normal-weight aggregate in the shooting process of the shotcrete.

## 1. Introduction

Shotcrete is a type of concrete mixed from coarse and fine aggregate, cement, admixtures, and other ingredients and then sprayed out through a spraying nozzle by high pressure or compressed air. For dry-mix shotcrete, water is sprayed out by another nozzle and mixed with the dry ingredients. For wet-mix shotcrete, water is mixed with the other ingredients of the concrete before the mixture is sprayed out [1,2]. Shotcrete has been widely used in mines, underground, in tunnels, and for structural reinforcement because of its advantages of short setting time, high early-age strength, and flexible operation mode [3,4].

In the construction process of shotcrete, factors such as selections of raw materials, mixture proportion design, construction quality control, and construction environment can affect the strength and durability of the resulting shotcrete. Large amounts of dust and high rebound rate (material waste) have been major issues for the construction of shotcrete, which can also cause shotcrete to have poor quality [5,6,7,8]. Although wet-mix shotcrete has shown better performance in reducing the rebound rate and dust than dry-mix, high rebound rate and dust amounts are still the major challenges that the shotcrete industry is facing.

Several studies have tried to improve the mechanical and durability properties and constructability (control of rebound rate) of shotcrete through adjustment of mixture proportion by adding admixtures, supplementary cementitious materials (SCM), fibers, nanomaterials, etc. One way is to use a blend of Poly naphthalene sulfonate superplasticizer and polyethylene oxide (PEO), which results in a significant boost in viscosity, thus reducing the rebound rate [9,10]. Yun et al. [11] investigated the effects of various admixtures, including silica fume (SF), air-entraining agent, superplasticizer, synthetic fiber, powdered polymer, and a viscosity agent, on the rheological properties of wet-mix shotcrete as an indicator of the rebound behavior of the shotcrete. Park et al. [12] added industrial by-product fly ash (FA) into shotcrete. The replacement rates of FA were 5–20%. From the results, the compressive strength of shotcrete decreased as FA content increased. Fly ash significantly reduced the charge passing capacity of shotcrete, indicating the improved compactness of the shotcrete. The shotcrete mixed with FA could also effectively reduce the penetration depth of chloride ions, thus resisting chloride ion erosion. Renan et al. [13] used blast furnace slag (BFS) to replace cement with a replacement rate between 0 and 30%. The results showed that, compared with that of shotcrete without BFS, the 1-day (1-d) compressive strength of BFS shotcrete was higher. A study by Maryam et al. [14] showed that adding nano-SiO_2_ and nano-Al_2_O_3_ to shotcrete resulted in better mechanical properties; adding 1.5% nano-SiO_2_ and 1% nano-Al_2_O_3_ improved the compressive strength and indirect tensile strength of shotcrete by 22.90% and 75%, respectively. In a study by Pan et al [6], when 3–7% accelerator and 1–3% tackifier were both applied to wet-mix shotcrete, the rebound rate of the shotcrete decreased. Furthermore, increasing the polypropylene fiber content resulted in first a decrease but later an increase in the rebound rate.

Coarse aggregates are the major rebound materials of shotcrete. Thus, it is critical and beneficial to reduce the rebound rate of coarse aggregates. Based on the theoretical model developed by Armelin and Banthia [15,16], a valid method for solving the issue of the high rebound rate of shotcrete is to use lightweight aggregate with lower density to replace the normal-weight coarse aggregate concrete in shotcrete [2,7,17,18,19]. Lightweight aggregate shotcrete (LAS) has the following advantages. The density of LAS ranges from 1500 to 1950 kg/m^3^, thus preventing the sprayed layer of shotcrete from falling off. In the sections where a tunnel needs to withstand high pressures and large deformation of the surrounding rocks and earth, a thicker shotcrete layer is needed, and LAS is preferable [20]. Previous studies have shown that lower density and smaller size of aggregate can effectively reduce the rebound of shotcrete [7,15,16,17]. Furthermore, saturated porous lightweight aggregate has been used as a method for the internal curing of concrete. During mixing, the aggregate can absorb partial water in the cement, thus reducing the local water/cement ratio in the interfacial transition zone (ITZ) and resulting in denser hardened cement [21,22].

Lightweight aggregate shotcrete is a type of lightweight aggregate concrete (LAC) with the features and functions of shotcrete. The density of LAC is lower than that of normal concrete, while the strength of LAC is not compromised. LAC has been widely used in industrial and residential buildings and bridge engineering because of its low density, high strength/weight ratio, low cost, good durability, excellent thermal insulation performance, and good fire resistance [23,24,25]. Numerous studies have been conducted on investigating the mechanical properties, bond strength, and durability of LAC when SCM, including fly ash, silica fume, and slag, is added [26,27,28,29,30,31,32,33,34]. Per a study by Chung et al. [35], the ITZ of LAC is different from that of normal concrete; there is no surface effect on the ITZ of LAC, so that the aggregate can form a better bond with the cementitious materials. It was also observed that the early strength of LAC developed rapidly, with a 7 d compressive strength (*fc′*) that reached 90% of the 28 d strength. These features are favorable to shotcrete. Maree and Riad [36] found a type of foamed LAC with higher bond strength than normal concrete.

The main types of lightweight aggregate are natural lightweight aggregate (such as cinder, pumice, etc.), industrial waste aggregate (such as fly ash ceramsite), and artificial lightweight aggregate (such as clay ceramsite, shale ceramsite, expanded perlite, etc.). In this study, a type of artificial lightweight shale ceramsite (ALSC) produced from industrial waste, which was porous but had high strength, was used as the coarse aggregate for lightweight aggregate shotcrete. ALSC is produced mainly from shale and other industrial waste as a type of green building material for making green LAC. It has the features of small dead weight, good thermal preservation, good energy conservation, and improved seismic performance. LAC made of ALSC has been applied in different types of industrial buildings and infrastructure, including thermal insulation pipes, thermal or noise barriers, tall buildings, and long-span bridges [37,38,39]. FA and SF were used to partially replace cement, and the effects of FA and SF on the properties of LAS were studied. Both experimental and theoretical studies were conducted to investigate the potential feasibility of using ALSC in lightweight aggregate shotcrete. From the experiments, the influences of SCMs on the mechanical properties and chloride ion penetration resistance of LAS were studied. Based on the model proposed by Armelin and Banthia [15], the rebound rate of ALSC in LAS was analyzed, thus proving the advantages of using ALSC as coarse aggregates in shotcrete.

## 2. Experiment

### 2.1. Raw Materials

Lightweight aggregate shotcrete is composed of lightweight coarse aggregate, fine aggregate, cement, SCM, and admixtures. Artificial lightweight shale ceramsite was used as the coarse aggregate. Fly ash and silica fume were used to partially substitute cement. Superplasticizer and accelerator were added to control the workability and the setting speed of shotcrete mixtures, respectively.

#### 2.1.1. Artificial Lightweight Shale Ceramsite

**Figure 1:**
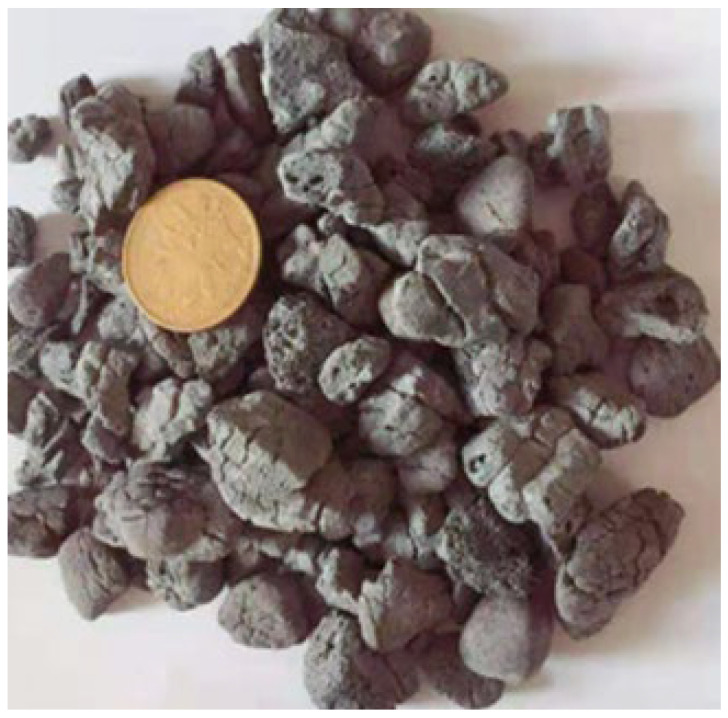
Artificial lightweight shale ceramsite with size of 5–15 mm.

Artificial lightweight shale ceramsite produced as shown in Figure 1 was used as the coarse aggregate for shotcrete. The raw materials for producing ALSC included municipal sludge, shale, fly ash, general clay, construction waste, remediation soil, etc. The raw materials were stored in a shed for natural drying and then sent to respective silos. Multiple roll-tooth crushers were used to crush clay into different particle sizes, which were then mixed with other raw materials (sludge, slag, fly ash, etc.) in a double-shaft mixer. After being stacked in the aging yard, the aged mixture was sent to an opposite roll granulator for extrusion and granulation. The produced granular material balls were sent to a shaping screening machine and screened out. The qualified granular balls were sent to a double-barrel plug-in rotary kiln for preheating and roasting. After the heated finished ceramsite products fell into the cooler for cooling, they were divided into sizes of 5 mm, 15 mm, and 25 mm and separately stored in the storage yard. Then, ceramsite of different sizes are bagged and delivered.

As shown in Table 1, the size of the ALSC ranged from 5 to 15 mm, and the apparent density of the ALSC was 1380 kg/m^3^.

#### 2.1.2. Cement

P.O. 42.5 cement (ordinary Portland cement with a strength grade of 42.5MPa) was used. The chemical composition is shown in Table 2. The physical and mechanical properties are shown in Table 3.

#### 2.1.3. Fine Aggregate

River sand with a fineness modulus of 2.8, apparent density of 2632 kg/m^3^, bulk density of 1630 kg/m^3^, and silt content of 1.6% was used as the fine aggregate of the shotcrete.

#### 2.1.4. Supplementary Cementitious Material

Fly ash and silica fume were used to partially replace cement in the shotcrete. Fly ash is an essential SCM used in concrete. It helps to reduce the consumption of cement, improve the workability of the concrete mixture through its lubricating role, reduce the hydration heat of mass concrete, and improve the durability of concrete because of its secondary hydration that consumes calcium hydroxide (CH). Grade I fly ash with a density of 2.059 g/cm^3^ was used.

Addition of SF in shotcrete can effectively improve the viscosity of concrete and the adhesion between the shotcrete and the sprayed surface. As a very active pozzolanic pore filler, SF can improve the compactness of concrete, which is beneficial to both the strength and durability of shotcrete. The specific surface area of the SF was 20 m^2^/g; the particle size was within 0.001–1 μm, with an average size of 0.12 μm; and the ignition loss was 3.72%. The main chemical components of both FA and SF are shown in Table 2.

#### 2.1.5. Admixtures

A polycarboxylic acid superplasticizer with a water reduction rate of 30% was used to improve the workability and sprayability of the shotcrete. A low alkali liquid accelerator was used to accelerate the setting time of the shotcrete.

### 2.2. Shotcrete Mixture Proportion

#### 2.2.1. Mixture Proportion Design

The specified 28 d compressive strength (*fc′*) of LAS was 25 MPa. The test plan for the mixture proportion of LAS is shown in Table 4. The water content, water/binder ratio (w/b), and cement replacement rate (CRR) of FA and the CRR of SF were selected as the main influencing factors in the mixture proportion design. The superplasticizer was applied to improve the workability of the mixture. Three levels of water content, 160 kg/m, 180 kg/m, and 200 kg/m^3^, were adopted. Based on the strength grade requirement of the shotcrete, the water/binder ratios were set as 0.42, 0.47, and 0.52. The CRRs of FA were set as 0%, 15%, and 30%, and the CRRs of SF were set as 0%, 5%, and 10%. The sand/total aggregate ratio is related to the consistency and viscosity of the shotcrete. Based on preliminary experimental investigations, the sand/total aggregate ratio was fixed at 56% [40].

#### 2.2.2. Casting Specimen for Mechanical Properties

Tests on the mechanical properties of the shotcrete specimens of the 9 mixture proportions shown in Table 4 were conducted. The tests for *fc′*, indirect tensile strength (*f_t_*), and bond strength (*τ*) of the specimens were included, and specimens were cast accordingly. Three 100 mm × 100 mm × 100 mm cubes for each mixture were tested for the compression and indirect tension (IDT) tests. For the bond strength test, the shotcrete was cast on the marble to form complete “half shotcrete–half marble” cubes. Through the IDT tests on these cubes, the bond strength was determined. During the mixing and casting, the accelerator was added after the concrete was evenly mixed. One day after casting, the specimens were demolded and under the standard curing. The density of the LAS was measured to be 1750 kg/m^3^.

### 2.3. Tests of Mechanical Properties

**Figure 2:**
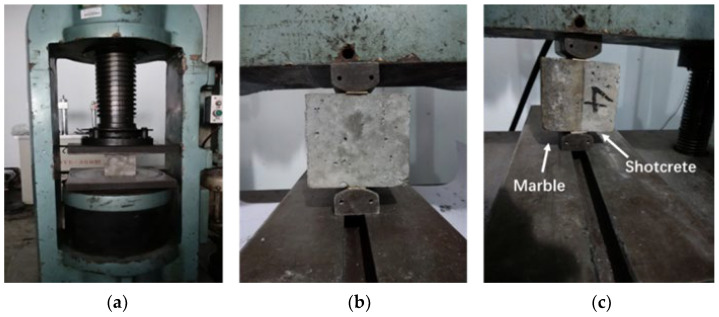
Mechanical tests on shotcrete cube specimens: (**a**) compression test; (**b**) indirect tension test; (**c**) bond strength test.

As shown in Figure 2a, a Nyl-600 compressive test machine was used to conduct the compression tests based on Standard GB/T50081-2019 [41]. As shown in Figure 2b,c, IDTs were conducted on both the shotcrete cubes and the shotcrete–marble cubes according to GB/T50081-2019 [41].

### 2.4. Tests on Chloride Ion Penetration Resistance

Influences of SCMs on the chloride ion penetration resistance (CPR) of LAS were studied through the electric flux test and rapid chloride migration (RCM) test. A total of 54 cylindrical specimens with diameters of 100 mm and heights of 50 mm were prepared based on GB-T50082-2009 [42], including casting, curing, waxing, and vacuum water retention, before the testing. Because the SCMs replace a large proportion of cementitious material, the curing period was extended from 28 d to 56 d to ensure that SCMs would hydrate sufficiently.

In the electric flux test, the CPR of concrete is reflected by the total amount of charge passing through in a certain period. The electric flux tests were conducted by using an IS-BSY electric flux acquisition system and according to ASTM C1202 [43].

The RCM test is based on the principle of electrochemical migration and according to the penetration depth of chloride ions and the chloride ion migration coefficient (*D_RCM_*) to evaluate the permeability of concrete against chloride ions, which is an indication of the durability of concrete. An RCM-NTB type chloride ion mobility coefficient analyzer manufactured by the Beijing NELD company was used. The specimens were first washed in an ultrasonic cleaning machine for 3 min and then installed in silicone sleeves. Then, 0.2 mol/l NaOH solution was injected into the silicone sleeve such that the specimen was immersed in the solution. Next, 5% NaCl solution was injected into the electrolytic cell, such that the liquid level of the electrolytic cell was equal to the liquid level in the sleeve. Then, the RCM test was conducted. After the test, the specimen was split into half along its midline, and the end face, immersed in NaOH solution, was sprayed with AgNO_3_ solution with a concentration of 0.1 mol/L to obtain the penetration depth of chloride ions (*x_d_*). Last, *D_RCM_* was calculated by Equation (1):

(1) $$D_{RCM}=2.872\times 10^{-6}\cdot \frac{T\cdot h\cdot \left(x_{d}-\alpha \cdot \sqrt{x_{d}}\right)}{t}$$

where:

*T* is the average of the initial and final temperature;
*h* is the actual height of the specimen;
*x_d_* is the chloride penetration depth;
*t* is the test time required for each specimen;
*α* = 3.338·10 ^−3^(*Th*) ^0.5^.

### 2.5. Contact Force Measurement

**Figure 3:**
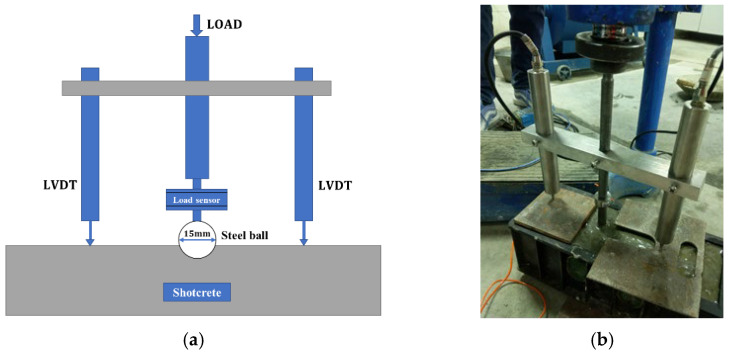
Measurement of contact force: (**a**) illustration of test setup; (**b**) test device.

Contact stress between the aggregate and the concrete occurs as the aggregate penetrates the fresh concrete. The test device for measuring the contact force, based on a study by Armelin and Banthia [15], is shown in Figure 3. Steel balls of the same diameter (15 mm) but with different densities were used to simulate coarse aggregates of different densities (593 kg/m^3^ for ALSC and 1578 kg/m^3^ for normal-weight aggregate). The load was gradually manually applied on the top of the rod such that the steel ball would penetrate the shotcrete mixture and the contact force between the aggregate and the mixture developed. A BSLM-3 type load sensor was connected to the steel ball through a steel rod to measure the contact force. Linear variable differential transformers (LVDT) were placed on both sides of the load sensor to measure the penetration depth.

Two mixture proportions with w/b fixed at 0.42 and sand/total aggregate ratio fixed at 0.56, one with ALSC and one with normal weight aggregate, were included. The contact forces were measured quickly after the accelerator was added to the shotcrete mixture.

## 3. Analyses of Test Results

Test results for *fc′* at different ages, 28 d *f_t_*, and 28 d *τ* of the LAS are shown in Table 5.

### 3.1. Compressive Strength of Lightweight Aggregate Shotcrete at Different Ages

**Figure 4:**
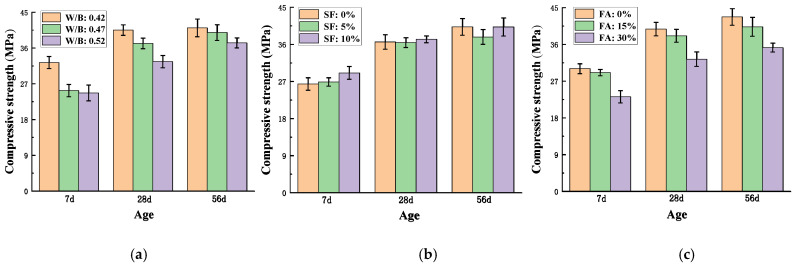
Compressive strength of lightweight aggregate shotcrete at different: (**a**) w/b ratios; (**b**) replacement rates of fly ash; (**c**) replacement rates of silica fume.

As shown in Figure 4, the compressive strength of LAS increased as age increased, while the increasing rate was reduced after the age of 28-d. The effects of w/b, the CRR of FA, and the CRR of SF on the *fc′* of LAS were analyzed.

As shown in Figure 4a, lower w/b resulted in higher *fc′* of LAS, as expected. When w/b was 0.42, 7 d *fc′* was 30% higher than that when w/b was 0.47 and 0.52, while at 28 d and 56 d, the differences in *fc′* between high and low w/b were much smaller. Furthermore, for higher w/b (0.47 and 0.52), the later strength increased more evidently, while *fc′* for w/b of 0.42 after 28 d almost did not increase. This was due to the accelerator, which contributed to the early strength development while sacrificing the later strength of the concrete. Furthermore, as the accelerator resulted in early strength development at a higher rate, most of the ultimate strength of the concrete had developed within 28 days, and only a slight increase in strength could develop afterwards [44,45]. This explains why the differences in 56 d *fc′* among different w/b were the smallest; the 56 d *fc′* of w/b = 0.42 was only 3% and 9% higher than those of w/c = 0.47 and 0.52, respectively. In summary, the effect of accelerator on the LAS made of ALSC was not compromised, as *fc′* of LAS increased rapidly at early age and slowed down at later age. Therefore, this type of ALSC is valid for use in shotcrete applications regarding the requirement of early strength development.

As shown in Figure 4b, *fc′* at 7 d, 28 d, and 56 d decreased as FA content increased. For the specimens with CRR of FA of 15%, *fc′* was slightly reduced; the *fc′* at 7 d, 28 d, and 56 d was 3%, 4%, and 4.8%, respectively, lower than that of the specimens without fly ash. When the CRR of FA reached 30%, the reduction effect on *fc′* became much more significant. This was consistent with past studies. The experimental results of Park et al. [12] showed that adding 5–20% FA led to reductions in the *fc′* of shotcrete. In a study by Mili et al. [46], adding 15–25% FA resulted in lower *fc′* of concrete. The activated silica and alumina in FA can react with CH from the hydration of cement, forming more C-S-H products that can fill the pores in concrete. However, the hydration degree of cement is low at early age, and the amount of CH is inadequate to react with FA. Therefore, partial replacement of cement with FA can result in reduction in the strength, and especially the early strength, of the concrete.

As shown in Figure 4c, the replacement ratio of SF had no significant effect on the *fc′* of LAS. At the age of 7 d, *fc′* increased slightly as the SF replacement rate increased. At age of 28 d, only at the CRR of SF = 10% did *fc′* increase slightly. At age of 56 d, little increase in *fc′* was shown as the content of SF increased in the mixture. The particles of SF are about two orders finer than those of cement and FA. Thus, SF is a good filler for concrete. Furthermore, SF contains highly active silica with a large specific surface area and can react with CH to reduce CH and form C-S-H gels. Consequently, the microstructure of concrete becomes more compacted, improving the strength and properties of the concrete. However, since ALSC is very porous, partial very fine SF particles might be absorbed in the pores during the mixing, weakening its effects of filling and secondary hydration.

### 3.2. Indirect Tensile Strength and Bond Strength

**Figure 5:**
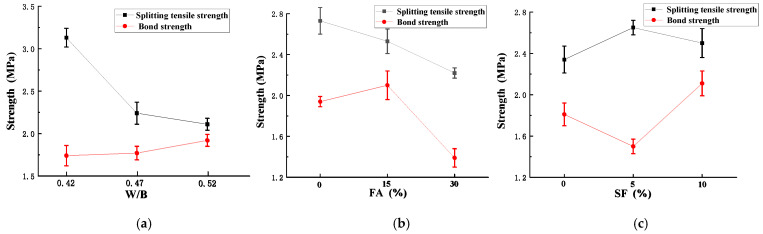
Indirect tensile strength and bond strength of LAS at different: (**a**) w/b ratios; (**b**) replacement rates of FA; (**c**) replacement rates of SF.

Test results for 28 d *f_t_* and *τ* are shown in Figure 5. The *f_t_* of LAS decreased as the w/b ratio decreased. As the w/b increased from 0.42 to 0.47, *f_t_* decreased significantly (29%), and the extent of the decrease grew smaller (6.9%) when w/b increased from 0.47 to 0.52. The highest *f_t_* of LAS was 3.13 MPa. On the other hand, as w/b decreased, *τ* increases. However, the increasing effect was slight, especially for w/b changes from 0.42 to 0.47.

As shown in Figure 5b, *f_t_* decreased linearly as FA content increased. When the CRR of FA was 15% and 30%, *f_t_* was 92% and 81%, respectively, of that of the control specimen (without fly ash). The increase in the CRR of FA indicated a reduction in cement content that could participate in the hydration. When the CRR of FA was 15%, the cement content, and thus the early strength, of the shotcrete decreased. However, when reaching the age of 28 d or older, the decrease in both *fc′* and *f_t_* of concrete due to the reduction in cement hydration was compensated by the secondary hydration of fly ash. However, if the CRR of FA further increased (30%), at the age of 28 d, the secondary hydration of FA was not completed, resulting in a more distinct decrease in the tensile strength. Therefore, it is more beneficial to keep the CRR of FA at –15%. The reductions in *f_t_* due to the addition of FA were consistent with past studies. In a study by Al-Yousuf et al. [47], in a concrete mixture of a strength grade of 32 MPa, as 25% FA was added, the 28 d tensile strength dropped from 4.03 to 3.83 MPa. Golewski [48] found that 30% FA resulted in reductions in the mechanical properties of concrete at early age, and improvements in strength were shown after the age of 6 months was reached.

The bond strength was significantly enhanced when the CRR of FA was 15%. When the CRR was 30%, *τ* decreased to 70% of that without fly ash. Therefore, the CRR of FA is suggested to be controlled at around 15% such that the decrease in *f_t_* is slight and *τ* increases.

As shown in Figure 5c, *f_t_* increased tortuously as SF increased; when the CRR of SF increased from 0% to 5%, *f_t_* increased slightly (0.2 MPa), and when the replacement ratio further increased from 5% to 10%, *f_t_* decreased slightly but was higher than that without SF. Generally, adding SF slightly improved the *f_t_* of lightweight aggregate shotcrete. This result was consistent with the observation from past studies that SF exerts insignificant influence on the splitting tensile strength of concrete [49,50,51]. For instance, Bhanja and Sengupt [52] replaced cement with SF in five concrete mixtures with different w/c ratios and found that SF either did not increase *f_t_* or increased *f_t_* insignificantly. The bond strength decreased when the CRR of SF is 5% but increased to a high level (17% higher than that without SF) when the CRR was 10%. Therefore, the CRR of SF is suggested to be controlled at 10% such that both *f_t_* and *τ* improve.

Based on the above analyses, considering the balances of the compressive strength, tensile strength, and bond strength of LAS, a mixture proportion of 180 kg/m^3^, w/b of 0.42, CRR of FA of ~15%, and CRR of SF of 10% is recommended.

### 3.3. Results of Electric Flux and Chloride Ion Migration Coefficient

Results of electric flux and DRCM are shown in Table 6. Note that as an SF CRR of 30% significantly reduced the strength of LAS, the CRR of SF for electric flux and RCM tests was adjusted to be 0%, 10%, and 20%. Furthermore, w/b was fixed at 0.42. For w/b of about 0.4, the typical electric flux of ordinary concrete is about 2000 coulomb, while the electric flux value of LAS without any SCM is 1252 coulomb. Moreover, the CPR of LAS is about 40% higher than that of ordinary concrete. These can be explained by the characteristics of water absorption and release of ALSC. During concrete mixing, ALSC fully absorbs water, which is slowly released during the curing, thus providing water for the unhydrated cement particles to further hydrate in the later age [53,54,55]. This mechanism results in a higher hydration degree of the LAS and thus a more densified and compacted microstructure of the mixture. Consequently, the chloride ion penetration resistance and the durability of the LAS are improved.

**Figure 6:**
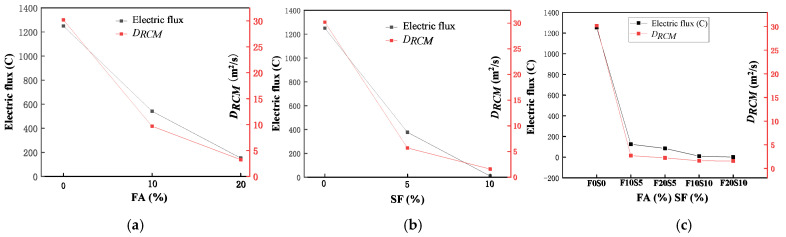
The resistance effects on chloride permeability of (**a**) different fly ash replacement rates; (**b**) different silica fume replacement rates; (**c**) different combinations of fly ash and silica fume replacement rates.

Effects of SCMs on the electric flux and *D_RCM_* of LAS are shown in Figure 6. As shown in Figure 6a, as FA content increased, both the electric flux and *D_RCM_* decreased, indicating improvement in the chloride ion permeability resistance of concrete; when the CRR of FA changed from 0% to 10%, the electric flux and *D_RCM_* decreased by 56% and 67%, respectively, and when CRR of FA increased from 10% to 20%, the electric flux and *D_RCM_* further decreased by 71% and 66%, respectively. This improvement was attributed to several mechanisms during the hydration of cementitious materials, including morphology effects, which can fill the capillary pores and pore cracks, and secondary hydration, which consumes CH and produces hydration products that fill the pores. In addition, FA has physical adsorption and chemical binding effects on chloride ions. FA has a vitreous micro-effect; the strong surface adsorption capacity and high surface tension of FA contribute to adsorbing the chloride ions in cement paste. At the same time, the high content of amorphous Al_2_O_3_ in FA reacts with chloride ions and forms Friedel salt, which can solidify chloride ions, thus improving the chloride ion permeability resistance of concrete [56,57].

As shown in Figure 6b, adding SF to LAS reduced the electric flux and *D_RCM_* values significantly; when 5% SF was added, the electric flux and *D_RCM_* were reduced by 70% and 81%, respectively, and when 10% SF was added, the electric flux value could not be measured because of the dense internal structure of the concrete, and the *D_RCM_* was reduced by 94%. Compared with FA, SF has a larger specific surface area that can fill the pores more sufficiently and is more reactive, with a better secondary hydration degree at 56 d, creating a denser microstructure of hardened concrete to resist chloride ion penetration. As shown in Figure 6c, when both FA and SF were added, the electric flux and *D_RCM_* were both improved compared with those when either FA or SF was added alone. This was because both SCMs have secondary hydration effects, and the particles are of different sizes, such that FA fills the pores in hydrated cement and SF fills the smaller pores that FA cannot. When SCM content reaches certain points (FA = 20% and SF = 5%-F20S5) with very low chloride ion permeability, there is no need to further increase FA content (FA = 20% and SF = 10%-F20S10), as this would only slightly increase the CPR but might sacrifice the strengths of the mixture.

## 4. Rebound Model of Shotcrete

The largest proportion of the materials of the entire rebounded mixture is from the rebounded coarse aggregate. Based on the rebound theory of shotcrete aggregate proposed by Armelin and Banthia [15,16], the movement of lightweight coarse aggregate during the shooting–penetration–rebound process of LAS was studied and analyzed in order to prove the validity of using LAS to reduce the rebound rate.

### 4.1. Rebound Theory of Shotcrete

The process from concrete being shot out to rebound can be divided into three stages, 1. flight phase; 2. penetration phase; and 3. reaction phase, based on the assumption that fresh shotcrete is an elastoplastic material that conforms to the Tresca yield criterion [15,16,17].

1. Flight Phase

A coarse aggregate of shotcrete, which is treated as a spherical projectile, has a certain initial velocity (*V*_0_) under the action of air pressure and flies along a parabola under *V_0_* and gravity [17]. The aggregate is also called the impactor, as it impacts the base material at the end of the flight phase and the start of the penetration phase.

2. Penetration Phase

When the aggregate reaches the shotcrete surface, it penetrates the base material and causes cavitation. A “hemispherical cavity” is assumed to form between the contact surfaces of the aggregate and the base material, which is under hydrostatic pressure-contact stress (*p*). During the further penetration, the stress state evolves into the elastoplastic and elastic states successively [15,17]. The relationships between the (cavity) radial stress (*σ_r_*) and *p* or the yield strength of the shotcrete mixture (*Y*) at different boundary conditions (BC) are summarized in Equations (2)–(4). The details of BC can be found in a study by Bindiganavile and Banthia [17].

(2) $$\sigma_{r}=p\;\;\;\;(r=a,\;\mathrm{under}\;\mathrm{hydrostatic}\;\mathrm{state})$$

(3) $$\frac{\sigma_{r}}{Y}=-2ln\left(\frac{c}{r}\right)-\frac{2}{3}\;\;\;(a<\;r<\;c,\;\mathrm{elastoplastic}\;\mathrm{region})$$

(4) $$\frac{\sigma_{r}}{Y}=-\frac{2}{3}ln{\left(\frac{c}{r}\right)}^{3}\;(r>\;c,\;\mathrm{elastic}\;\mathrm{region})$$

where:

*σ_r_* is the radial stress;
*p* is the contact stress in hydrostatic state;
*a* is the radius of the contact area between the aggregate and the base material;
*r* is the radius of the cavity;
*c* is the radius of the elastic boundary around hydrostatic core;
*Y* is the yield strength of the shotcrete mixture.

Based on the theory of plasticity and the compatibility requirements of BC, *p* at different locations during the entire penetration process was calculated by Equation (5) [15,17,58].

(5) $$\frac{p}{Y}=\frac{2}{3}\left\{ln\left(\frac{aE}{3RY}\right)+1\right\}$$

where:

*E* is the modulus of elasticity of fresh shotcrete;
*R* is the radius of the aggregate, which is assumed as a spherical projectile.

A dynamic contact stress (*p_d_*) on the aggregate surface is developed during shotcrete spraying. When the aggregate penetrates the base material, *p_d_* is assumed to be constant in the contact area. Therefore, as shown in Equation (6), the work done by *p_d_* in the penetration process (*W*_1_) is equivalent to the kinetic energy of coarse aggregate under the impact kinetic energy [15,17]:

(6) $$\frac{1}{2}mV_{0}{}^{2}=W_{1}=p_{d}v_{c}$$

where:

*v_c_* is the volume of the cavity;
*p_d_* is the dynamic contact stress;
*m* is the mass of the impactor (aggregate);
*V*_0_ is the initial speed of the impactor (aggregate).

3. Reaction Phase

As the aggregate further penetrates the shotcrete base material in the elastic region, the strain energy is accumulated in both the aggregate and the base material. It is finally released into the aggregate, causing a rebound—the “reaction phase”.

Based on Tabor’s method for calculating the rebound energy of metal balls impacting a metal base and Hertz contact conditions, the rebound energy of shotcrete (*W*_2_) can be expressed as Equation (7) [15,17,59]:

(7) $$W_{2}=\frac{3\pi^{2}}{10}\cdot p^{2}\cdot {\left(a^{*}\right)}^{3}\cdot \left(\frac{1-\mu_{c}{}^{2}}{E_{c}}+\frac{1-\mu_{i}{}^{2}}{E_{i}}\right)$$

where:

*E_c_* and *µ_c_* are the modulus of elasticity and Poisson’s ratio of the (shotcrete) base material, respectively;
*E_i_* and *µ_i_* are the modulus of elasticity and Poisson’s ratio of the impactor (coarse aggregate), respectively;
a* is the maximum contact area radius between the aggregate and the base material in the penetration stage.

#### 4.1.1. Influencing Factors of Coarse Aggregate Rebound

The difference between *W_1_* and *W_2_* determines the rebound behavior of the aggregate. As shown in Equation (8), the rebound coefficient (*e*) is equal to the ratio of impact velocity (*V_i_*) to rebound velocity (*V_r_*). Note that no energy loss is assumed during the flight phase, so *V_i_* is equal to the initial velocity, *V_0_*. A larger aggregate rebound coefficient indicates a greater rebound rate of the aggregate. Substituting Equations (6) and (7) into Equation (8), ignoring 1/*E_i_* in the equation (*E_i_* is several orders higher than that of fresh concrete), *e* is expressed and simplified as Equation (9) [15,17]. Therefore, the main factors affecting aggregate rebound are the static contact stress, dynamic contact stress, and impact velocity.

(8) $$e=\frac{V_{r}}{V_{i}}=\frac{V_{r}}{V_{0}}=\sqrt{\frac{W_{2}}{W_{1}}}$$

(9) $$e=\left(\sqrt{\frac{3\pi^{2}}{10}\cdot {\left(\frac{4R}{\pi }\right)}^{\frac{3}{4}}\cdot \frac{1}{E_{c}}\cdot {\left(\frac{1}{2}m\right)}^{-\frac{1}{4}}}\right)\cdot p\cdot {\left(p_{d}\right)}^{-\frac{3}{8}}\cdot {\left(V_{0}\right)}^{-\frac{1}{4}}$$

#### 4.1.2. Influencing Factors of Rebound of Shotcrete with Different Aggregate Densities

Considering separating the constant part (*K*) and the influencing part grouped with variables (*ψ*), named the “influence factor”, in Equation (9), Armelin and Banthia [15] proposed an expression of e as shown in Equation (10) and provided the expressions of *K* and *ψ* as well. In Armelin and Banthia’s expressions, the mass of the impactor is treated as a constant and included in K parameter [15]. However, the coarse aggregates come in different sizes and materials, precisely, different volumes (*V_a_*) and densities (*ρ*). Thus, *V_a_* and *ρ* should be both included in the *e* parameter. As impactors of the same volume are commonly used in experimental studies, Bindiganavile and Banthia [17] further adjusted the expressions of *K* and *ψ,* in which *ρ* is included in *ψ* and *V_a_* is included in *K*. When the power of the air compressor for shooting shotcrete was constant, the *V_o_* of lightweight aggregate was higher than that of normal-weight aggregate of the same volume [17]. Furthermore, concrete made of aggregates with different densities might have different moduli of elasticity. Thus, *E_c_* is treated as a variable, and the expressions of *K* and *ψ* are modified accordingly as shown in Equations (10) and (11). Therefore, the influencing factors of rebound of shotcrete with different aggregate densities include the aggregate density, static and dynamic contact stress, initial aggregate velocity (caused by different densities), and elastic modulus of fresh concrete.

(10) e=K⋅ψ

where:

*K* is a constant;
*ψ* is the influence factor of coarse aggregate rebound.
  (11) $$K=\sqrt{\frac{3\pi^{2}}{10}\cdot {\left(\frac{4R}{\pi }\right)}^{\frac{3}{4}}\cdot {\left(\frac{1}{2}v_{a}\right)}^{-\frac{1}{4}}}$$
  (12) $$\Psi =\rho^{-\frac{1}{8}}\cdot p\cdot {\left(p_{d}\right)}^{-\frac{3}{8}}\cdot {\left(V_{0}\right)}^{-\frac{1}{4}}\cdot \sqrt{\frac{1}{E_{c}}}$$
  where:

*ρ* is the density of the impactor.

### 4.2. Analysis of Influencing Factors on Rebound of Aggregate with Different Density

#### 4.2.1. Contact Stress

**Figure 7:**
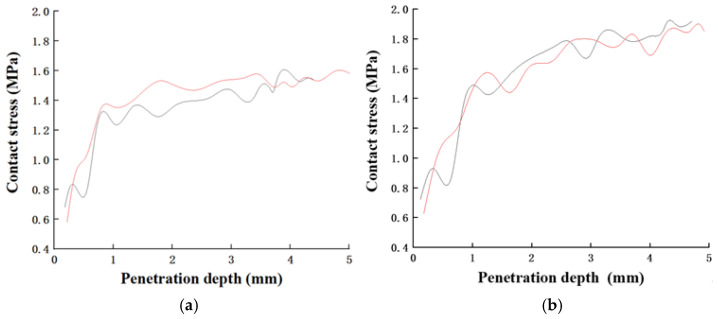
Contact stress for steel balls of different densities at different penetration depths in mortar: (**a**) 593 kg/m^3^ to represent lightweight aggregate; (**b**) 1578 kg/m^3^ to represent normal weight aggregate. (Two lines represent the two repeats per scenario).

As shown in Figure 3, the contact load and the penetration depth of the steel ball, which was used to represent coarse aggregate, into the mixture were measured. The contact stress of shotcrete between coarse aggregate (steel ball) and the mixture was obtained by dividing the applied load by the contact area based on the penetration depths. Then, the relationship between the contact stress and the penetration depth was obtained as shown in Figure 7. The trends of the curves of two different aggregate densities were consistent. Initially, the contact stress increased significantly as the penetration depth increased. When the penetration depth reached a certain value (~1 mm), the rate at which contact stress increased dropped, which was consistent with Armelin’s findings [15]. The contact stress of LAS was about 1.6 MPa, which was 20% smaller than that of ordinary shotcrete, with a contact stress of about 2.0 MPa.

#### 4.2.2. Dynamic Contact Stress

According to the initial kinetic energy of aggregate, the dynamic contact stresses of lightweight aggregate (p_d_*) and normal aggregate (*p_d_*) can be calculated by Equations (13) and (14), respectively:

(13) $$p_{d}^{*}\cdot v_{a}^{*}=\frac{1}{2}\cdot m^{*}\cdot {\left(V_{0}^{*}\right)}^{2}$$

(14) $$p_{d}\cdot v_{a}=\frac{1}{2}\cdot m\cdot V_{0}^{2}$$

#### 4.2.3. Modulus of Elasticity

The modulus of elasticity of fresh shotcrete, *E_c_*, cannot be tested directly. Instead, the modulus of elasticity of shotcrete after adding accelerator for 16 h (*E_c_*′) was tested to represent *E_c_*. The specimen size was 100 × 100 × 300 mm. *E_c_*′ for two mixture proportions with w/b fixed at 0.42 and sand/total aggregate ratio fixed at 0.56, one with ALSC and one with normal weight aggregate, was tested. It was found that the *E_c_*′ of both types of shotcrete at 16 h was almost equivalent; that of LAS was 19.0 GPa, and that of ordinary shotcrete was 19.2 GPa. Therefore, the effect *E_c_* on the differences in rebound rate between the LAS and ordinary shotcrete can be ignored. However, this observation applies only to the case in this study.

#### 4.2.4. Initial Velocity

As described earlier, the *V*_0_ of aggregates with different densities is different. Bindiganavile and Banthia [17] used a high-speed camera to record the time-of-flight phase for aggregates of different particle diameters and densities and calculated *V*_0_ accordingly, thus establishing the relationships between *V*_0_ and the *ρ* of aggregates. The relationship for the aggregate diameter of 12.7 mm is shown in Equation (15). The densities of 593 kg/m^3^ and 1578 kg/m^3^ (normal weight aggregate) were put into Equation (15) to calculate *V*_0_ for the two types of aggregates. These were 43.56 m/s for ALSC and 16.48 m/s for normal-weight aggregate.

(15) $$V_{0}=26007.34\cdot \rho^{-1}$$

#### 4.2.5. Influence Factor of Rebound

Based on the above results and according to Equation (12), the *ψ* of the two aggregate densities was calculated and compared as shown in Equation (16). It was found that the *ψ* of lightweight aggregate was about half of that of normal weight aggregate. This indicates that, compared with ordinary shotcrete, lightweight aggregate shotcrete using ALSC could result in a much weaker rebound trend and thus lower rebound rate of aggregate. The above theoretical analyses explain the mechanism of how using lightweight aggregates can significantly reduce the rebound rate compared with using normal-weight aggregates. However, on-site experiments or practices are needed to verify this conclusion.

(16) $$\frac{\Psi^{*}}{\Psi }=\frac{{\left(\rho^{*}\right)}^{-\frac{1}{8}}\cdot p^{*}\cdot {\left(p_{d}^{*}\right)}^{-\frac{3}{8}}\cdot {\left(V_{0}^{*}\right)}^{-\frac{1}{4}}\cdot \sqrt{\frac{1}{E_{c}^{*}}}}{\rho^{-\frac{1}{8}}\cdot p\cdot {\left(p_{d}\right)}^{-\frac{3}{8}}\cdot {\left(V_{0}\right)}^{-\frac{1}{4}}\cdot \sqrt{\frac{1}{E_{c}}}}\approx 0.5$$

## 5. Conclusions

Investigations of lightweight aggregate shotcrete (LAS) using artificial lightweight shale ceramsite (ALSC) as the coarse aggregate were conducted through both experimental approaches and theoretical analyses. Both fly ash (FA) and silica fume (SF) were used to partially replace cement to investigate their effect on the mechanical properties of the resulting shotcrete, including compressive strength (*f_c_’*), indirect tensile strength (*f_t_*), and bond strength (*τ*), as well as on the chloride ion penetration resistance as an indication of compactness and durability. Theoretical analyses on the rebound trend of LAS were conducted. The conclusions were drawn as follows:

(1) As expected, the water/cementitious material (w/b) ratio had a significant influence on the *f_c_’* and *f_t_* of LAS; the lower the w/b ratio was, the higher the *f_c_’* and *f_t_* of the LAS mixture were. When the w/b ratio decreased from 0.52 to 0.47 and 0.42, only an extremely slight decrease in *τ* was observed. Therefore, it is beneficial to keep a low w/b ratio for LAS, as *f_c_’* and *f_t_* are improved and *τ* is only negligibly sacrificed.
(2) As the cement replacement rate of FA increased (to 15% and 30%), both the *f_c_’* and the *f_t_* of the LAS decreased evidently, while *τ* first increased slightly at FA = 15% but decreased drastically at FA = 30%, which is undesired for shotcrete. Therefore, the replacement rate of FA is suggested to be controlled at near 15%.
(3) As the cement replacement rate of SF increased (to 5% and 10%), *f_c_’* increased accordingly, and *f_t_* first increased and then slightly decreased, albeit to a level still higher than that without adding SF content. Furthermore, *τ* decreased at SF = 5% but increased significantly at SF = 10%. Therefore, the replacement rate of SF is suggested to be controlled at 10%.
(4) Based on the results of the electric flux test and rapid chloride migration (RCM) test on the LAS, replacing cement with SF and FA significantly increased the chloride ion penetration resistance of (CPR) of LAS, indicating that the permeability of the mixture was reduced.
(5) Coarse aggregate made up the majority part of the rebound materials of shotcrete. Based on the rebound model of shotcrete, the main influencing factors of the rebound rate of coarse aggregate included aggregate density, static and dynamic contact stress, initial aggregate velocity, and the elastic modulus of fresh concrete. These factors were represented by a rebound coefficient. Through experiments and mathematical models, the above influencing factors of both ALSC and normal-weight aggregate were obtained and used to calculate the rebound coefficient. The rebound coefficient of ALSC for LAS was about half of that of the normal-weight aggregate for ordinary shotcrete, thus proving the feasibility and benefit of using ALSC as the coarse aggregate in shotcrete.

## Figures and Tables

**Table 1:** Basic parameters of artificial lightweight shale ceramsite.

Passing % (Sieve Analysis)				Apparent Density (kg/m^3^)	Packing Density (kg/m^3^)	Compressive Strength (MPa)
20 mm	15 mm	9.5 mm	4.75 mm			
0.8	45.8	51.7	1.7	1380	683	4.2

**Table 2:** Components in P.O. 42.5 cement, fly ash, and silica fume used for shotcrete.

Chemical Component	SiO_2_	Al_2_O_3_	Fe_2_O_3_	CaO	MgO	SO_3_	Na_2_O	f-CaO
Cement	21.75	4.6	3.46	64.54	3.56	0.46	0.6	0.96
Fly Ash	51.24	26.99	14.72	0.09	0.18	2.16	0.23	
Silica Fume	91.27	0.17	0.45	0.45	0.92			

**Table 3:** Physical and mechanical properties of P.O. 42.5 cement used for shotcrete.

Passing 0.08 mm (%)	Density (g/cm^3^)	Specific Surface Area (m^2^/kg)	Normal Consistency (%)	Stability (Le Chatelier Method) (mm)	Setting Time (min)	Setting Time (min)	Flexural Strength (MPa)		Compressive Strength (MPa)	
					Initial	Final	3 d	28 d	3 d	28 d
0.6	3.14	349	25.0	0.5	151	210	5.7	8.8	28.5	51.1

**Table 4:** Mixture proportion of shotcrete for testing mechanical properties.

No. of Mixture	Water Content (kg/m^3^)	Cementitious Material (kg/m^3^)	Replacement Rate of FA (%)	Replacement Rate of SF (%)	Fine Aggregate (kg/m^3^)	Coarse Aggregate (kg/m^3^)	Accelerator (%)	Superplasticizer (%)
1	160	381.0	0	0	560	440	1.5	3.0
2	160	340.4	15	5	560	440	1.5	3.0
3	160	307.7	30	10	560	440	1.5	3.0
4	180	428.6	30	5	560	440	1.5	3.0
5	180	383.0	0	10	560	440	1.5	3.0
6	180	346.2	15	0	560	440	1.5	3.0
7	200	476.2	15	10	560	440	1.5	3.0
8	200	425.5	30	0	560	440	1.5	3.0
9	200	384.6	0	5	560	440	1.5	3.0

**Table 5:** Average test results of mechanical properties of lightweight aggregate shotcrete.

No. of Mixture	7 d Compressive Strength (MPa)	28 d Compressive Strength (MPa)	56 d Compressive Strength (MPa)	28 d Indirect Tensile Strength (MPa)	28 d Bond Strength (MPa)
1	33.6	43.4	44.3	3.13	2.01
2	26.1	38.2	38.3	2.36	1.89
3	21.8	28.7	33	1.76	1.93
4	26.5	36.7	35.7	2.83	1.06
5	28.5	41.5	44.4	2.3	2.25
6	24.3	34.6	39.4	1.81	2.27
7	36.9	41.5	43.3	3.43	2.15
8	21.2	31.7	37	2.07	1.17
9	28	34.3	39.5	2.77	1.57

**Table 6:** Results of electric flux and chloride migration coefficient.

No.	w/b	Fly Ash (%)	Silica Fume (%)	Penetration Depth (mm)	DRCM (m^2^/s)	Electric Flux (C)	Permeability
1	0.42	0	0	50	30.18	1252	low
2	0.42	10	0	21.75	9.66	542	Very low
3	0.42	20	0	14.55	3.24	153	Very low
4	0.42	0	5	18.4	5.7	377	Very low
5	0.42	10	5	13.8	2.7	125	Very low
6	0.42	20	5	11.2	2.23	85	Extremely low
7	0.42	0	10	10.6	1.7	Too small, ignored	Extremely low
8	0.42	10	10	9.6	1.61	Too small, ignored	Extremely low
9	0.42	20	10	8.7	1.56	Too small, ignored	Extremely low
